# Regulation of Macrophage Activation and Polarization by HCC-Derived Exosomal lncRNA TUC339

**DOI:** 10.3390/ijms19102958

**Published:** 2018-09-28

**Authors:** Xue Li, Yi Lei, Miao Wu, Nan Li

**Affiliations:** 1 Department of Immunology, West China School of Basic Medical Sciences & Forensic Medicine, Sichuan University, Chengdu 610041, China; 18215530853@163.com (X.L.); miaowu0506@163.com (M.W.); 2 Department of Physiology, West China School of Basic Medical Sciences & Forensic Medicine, Sichuan University, Chengdu 610041, China; 17396230615@163.com

**Keywords:** exosomes, macrophage, lncRNA, inflammation, polarization

## Abstract

Exosomes released by cells can serve as vehicles for delivery of biological materials and signals. Long non-coding RNAs (lncRNAs) are non-coding RNAs longer than 200 nt, which roles are increasingly appreciated in various biological content. Tumor-derived exosomal lncRNAs have been implicated as signaling mediators to orchestrate cell function among neighbor tumor cells. However, the role of tumor-derived lncRNAs in cross-talk with environmental macrophages has yet to be explored. In this paper, we demonstrated that hepatocellular carcinoma (HCC) cells–derived exosomes contain elevated levels of lncRNA TUC339 and that HCC-derived exosomes could be taken up by THP-1 cells. In seeking to dissect the biological function of tumor secreting TUC339 in macrophages, we applied loss-of-function and gain-of-function strategies. We observed increased pro-inflammatory cytokine production, increased co-stimulatory molecule expression, and enhanced phagocytosis upon suppression of TUC339 by siRNA in THP-1 cells, and the opposite effect upon over-expression of this lncRNA, which indicates that TUC339 was involved in the regulation of macrophage activation. Moreover, we detected an elevated level of TUC339 in M(IL-4) macrophages as compared to M(IFN-γ + LPS) macrophages and a down-regulation of TUC339 expression during M(IL-4)-to-M(IFN-γ + LPS) repolarization and vice versa. Furthermore, suppression of TUC339 in macrophages diminished the expression of M(IL-4) markers upon IL-4 treatment while overexpression of TUC339 in macrophages enhanced M(IL-4) markers upon IFN-γ + LPS treatment, which suggests a critical function of TUC339 in the regulation of macrophage M1/M2 polarization. Lastly, using microarray analysis, we identified cytokine-cytokine receptor interaction, CXCR chemokine receptor binding, Toll-like receptor signaling, FcγR-mediated phagocytosis, regulation of the actin cytoskeleton, and cell proliferation are related with TUC339 function in macrophages. Our results provide evidence for a novel regulatory function of tumor-derived exosomal lncRNA TUC339 in environmental macrophages and shed light on the complicated interactions between tumor and immune cells through exosomal lncRNAs.

## 1. Introduction

Long non-coding RNAs (lncRNAs) are RNAs that are longer than 200 nucleotides and do not code for proteins [1]. Thousands of lncRNAs have been discovered to date. By binding to DNA, mRNAs, miRNAs or target proteins, lncRNAs can regulate the transcription, processing, translation, turnover, and protein activity of other protein coding genes [2]. LncRNAs are found to play important roles in regulating genomic imprinting, cell cycle, cell differentiation, tumorgenesis, metastasis and a variety of biological processes [3,4,5]. Accumulating evidence also emerge to suggest lncRNA function in both innate and adaptive immunity including their regulation of the development, activation, and homeostasis of the immune system [6].

Exosomes are small extracellular vesicles and are composed of a lipid bilayer ~50 to 100 nm in diameter. They are secreted from multivesicular endosomes by many different cell types and exist in almost all biological fluids as well as in culture medium of most cell types [7]. Exosomes contain a large variety of biological components including proteins, mRNAs, miRNAs [8], and lncRNAs [9] and hold great promise as novel biomarkers for clinical diagnosis. Both in vivo and in vitro studies have demonstrated that exosomes can be taken up by target cells and play important roles in cell-cell communication by transferring the molecular constituents to target cells [10]. A substantial body of literature has implicated exosomes–mediated transfer of lncRNAs in regulating immune response, cellular stress responses, chemosensitivity, tumor cell growth, and adhesion [11,12,13,14].

Macrophages are critical players in the innate immune defense and are increasingly appreciated as participants in the tumor microenvironment. Macrophages are a functionally heterogeneous cell population that hold a high degree of plasticity. In response to stimulation in the microenvironment, macrophage can be phenotypically polarized to two main groups: classical (or M1) and alternative (or M2) macrophages. M1 macrophages, which can be induced in vitro by lipopolysaccharide (LPS) and interferon γ (IFN-γ) elicit rapid proinflammatory responses, pathogen clearance, and display anti-tumor activity [15]. M2 macrophages, which can be induced in vitro by interleukin 4 (IL-4) and IL-13 show anti-inflammatory and tissue reparative activities, can promote tumorgenesis and tumor progression [16]. The switch from a proinflammatory (M1) to an anti-inflammatory phenotype (M2) is usually observed in tumor-associated macrophages during tumor progression from the early stage to a more advanced stage [17].

Previously, lncRNA TUC339 was identified and found to be enriched in HCC-derived exosomes [18]. Exosomal transfer of TUC339 to neighbor tumor cells promotes HCC cell proliferation and reduces cell adhesion to extracellular matrix. In this case, we ask whether HCC-derived exosomal lncRNAs can be transferred to neighbor macrophages and how this intercellular communication would influence macrophage function. We show that HCC-derived exosomes can indeed target neighbor macrophages and subsequently transfer TUC339 and other lncRNAs to macrophages. Gain and loss-of-function studies suggest TUC339 modulates macrophage cytokine production, phagocytosis, and M1/M2 polarization. These findings provide new mechanistic insights into exosomes as mediators in the tumor microenvironment and lncRNAs act as modulators of macrophage polarization, which advances our knowledge towards a tumor-immune interaction.

## 2. Results

### 2.1. Extraction and Morphological Examination of Hepatocellular Carcinoma Cell (HCC)-Derived Exosomes

**Figure 1:**
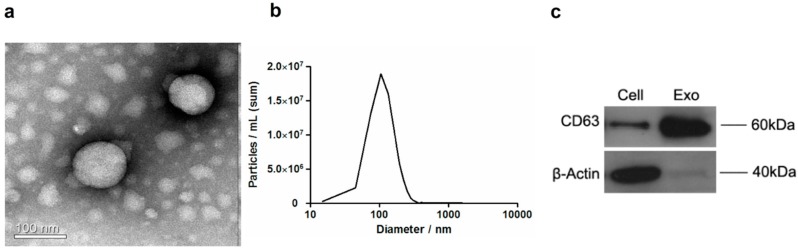
Characterization of HCC-derived exosomes. (**a**) A representation Transmission electron microscope (TEM) image of isolated exosomes. (**b**) Nanoparticle tracking analysis of isolated exosomes. The histogram shows the distribution of the size of vesicles. (**c**) Western blotting analysis of CD63 expression in both cell lysate and extracted exosomes from PLC/PRF/5 cells.

In order to study the impact of HCC-derived exosomes on macrophages, we first extracted exosomes from PLC/PRF/5 cells by differential ultracentrifugation. To verify the effectiveness of the isolation procedure, we examined the morphological appearance of the extracted exosomes by transmission electron microscopy. As shown in Figure 1a, the vesicles were found spherical and homogeneous in size (Figure 1a). Nanoparticle tracking analysis (NTA) showed that the diameters of exosomes mainly distribute around 100 nm with a range from 60 to 250 nm (Figure 1b). Furthermore, the expression of CD63, which is a commonly used exosomal marker, was detected in the extracted exosomes by Western blotting. The characteristics of morphology, size distribution, and biomarker expression were all consistent with previous reports for exosomes, which indicates successful enrichment of exosomes [19]. We, therefore, adopted this exosomal extraction approach for the rest of the study.

### 2.2. PLC/PRF/5-Derived Exosomes Can Be Internalized by THP-1 Cells

**Figure 2:**
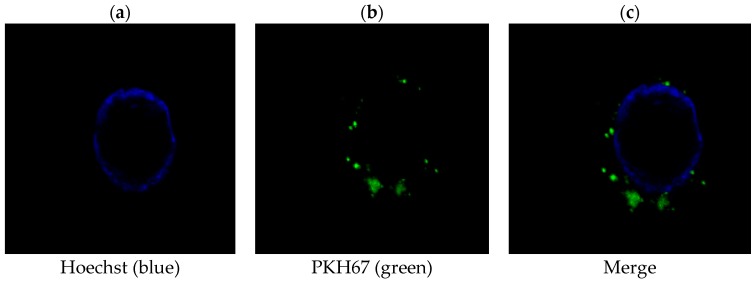
Internalization of HCC-derived exosomes by THP-1 cells. Macrophages were incubated with PLC/PRF/5-derived exosomes and labeled with PKH67 green dye for 24 h. Cell nuclei were stained by Hoechst (blue). Images were obtained using a confocal fluorescent microscope under 60x oil lens. (**a**) Hoechst (blue), (**b**) PKH67 (green), and (**c**) Merge. PLC/PRF/5-derived exosomes are shown to be internalized into the cytoplasm of macrophages.

Next, we asked whether HCC-derived exosomes could possibly target macrophages in the environment. We labeled the collected exosomes from PLC/PRF/5 with green fluorescent dye PKH67 and incubated THP-1 cells with labeled exosomes for 24 h. After a brief nucleus staining with Hoechst 33258, THP-1 cells were visualized using confocal microscopy to examine the relative localization of exosomes to the cells. As seen in Figure 2, exosomes were found inside THP-1 cells spreading all over the cytoplasm with increased perinuclear accumulation. This result strongly suggests PLC/PRF/5-derived exosomes can be internalized by THP-1 cells.

### 2.3. Long Non-Coding RNA TUC339 is Enriched in PLC/PRF/5-Derived Exosomes

**Figure 3:**
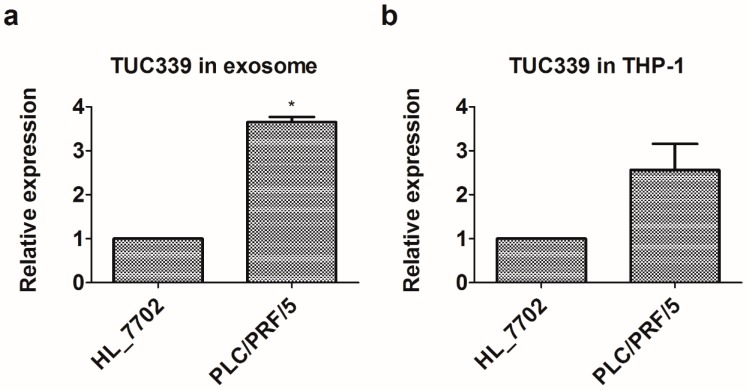
Enrichment of TUC339 in HCC-derived exosomes and subsequent transfer to THP-1 cells. (**a**) TUC339 is enriched in PLC/PRF/5-derived exosomes as compared to HL-7702 derived exosomes. (**b**) Upon incubation with supernatant of PLC/PRF/5 cells, THP-1 expressed elevated level of TUC339. Bars represent the mean ± SEM of three separate determinations. * *p* < 0.05.

Previous studies suggested that HCC-derived exosomes contain lncRNAs and several lncRNAs are elevated in exosomes isolated from HCC as compared to that from the normal liver cell line [20]. In order to verify this, we isolated exosomes from both HCC PLC/PRF/5 and a normal liver cell line HL-7702. Total RNAs were extracted from exosomes and the expression level of lncRNA-TUC339, lincRNA-VLDLR were analyzed using qRT-PCR. Both lncRNAs tested showed increased expression in exosomes from HCC as compared to that from a normal liver cell (Figure 3a). A greater increase was observed in TUC339—a more than three-fold increase in PLC/PRF/5 exosomes compared to HL-7702 exosomes. Enrichment of these lncRNAs in HCC-derived exosomes indicates their potential function as an important intercellular mediator.

Since PLC/PRF/5-derived exosomes can be internalized by THP-1 cells and PLC/PRF/5-derived exosomes carry enriched amount of TUC339, we, therefore, reasoned that PLC/PRF/5 cells can deliver TUC339 to neighbor THP-1 cells more than HL-7702 cells do. In order to confirm this, we cultured PLC/PRF/5 and HL-7702 cells to the same confluency, then collected the same amount of culture medium from both cell cultures and transferred supernatants onto THP-1 cells, respectively. After 24 h of incubation, total RNAs were isolated from THP-1 cells. Endogenous TUC339 was quantified by qRT-PCR. As seen in Figure 3b, we found THP-1 cells treated with PLC/PRF/5 supernatant expressed an elevated level of TUC339 than treated with an HL-7702 supernatant. This result suggests HCCs can deliver TUC339 to neighbor THP-1 cells more than normal liver cells do. We, thus, wonder the biological function of these HCC-secreted lncRNAs in the following studies by focusing on TUC339.

### 2.4. Knockdown of TUC339 in THP-1 Cells Leads to Increased Pro-Inflammatory Cytokine Production, Increased co-Stimulatory Molecule Expression, Enhanced Phagocytosis, and Reduced Viability

**Figure 4:**
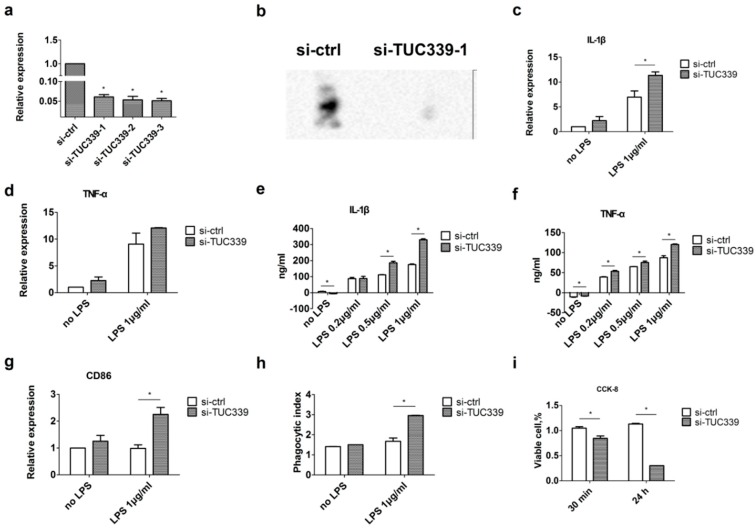
Knockdown of TUC339 in THP-1 cells leads to increased pro-inflammatory cytokine production, increased co-stimulatory molecule expression, enhanced phagocytosis, and reduced viability. THP-1 cells were transfected with siRNAs against TUC339 or unrelated siRNA control. Both (**a**) qRT-PCR and (**b**) Northern blot analysis showed effective knockdown of TUC339 by siRNAs. Upon TUC339 knockdown, IL-1β (**c**) and TNF-α (**d**) mRNAs were elevated in THP-1 cells, which is shown by qRT-PCR. IL-1β (**e**) and TNF-α (**f**) secretion were elevated, which is shown by ELISA. (**g**) CD86 mRNA was elevated as shown by qRT-PCR. (**h**) Phagocytosis was enhanced in LPS challenged THP-1 cells upon TUC339 knockdown. (**i**) Cell viability was reduced in THP-1 cells upon TUC339 knockdown, which is shown by the CCK-8 assay. Data represent mean ± SEM of three independent experiments. * *p* < 0.05.

Biological function of lncRNAs in HCC-derived exosomes has not been fully understood. Previous studies revealed a pro-proliferation and pro-metastasis function of TUC339 when transferring to adjacent HCCs [18]. Their impacts on other cell types in the microenvironment have not been investigated. Since TUC339, lincRNA-VLDLR containing exosomes are capable of being internalized by neighbor macrophages, we asked what would be the effect of these lncRNAs on environmental macrophages. To address this question, we adopted lost-of-function and gain-of-function strategies. We first transfected siRNAs targeting either TUC339 or lincRNA-VLDLR to THP-1 cells. As seen by qRT-PCR (Figure 4a) and Northern blotting (Figure 4b), TUC339 expression was significantly decreased in THP-1 cells upon incubation with any of three distinct siRNAs when compared to non-targeting siRNA control. This result strongly indicates TUC339 was successfully knocked down in a sequence specific manner. Similarly, qRT-PCR results show lincRNA-VLDLR can be successfully knocked down by corresponding siRNAs (Figure S1b).

Next, we investigated the effect of lncRNA knockdown on cytokine production in macrophage cells. THP-1 cells were first transfected with either lncRNA siRNA or control siRNA. Then mRNA levels of two key pro-inflammatory cytokines IL-1β and TNF-α were measured by qRT-PCR with or without LPS stimulation. Compared to control siRNA, we found an increase in IL-1β and TNF-α mRNA expression upon TUC339 knockdown with or without LPS stimulation (Figure 4c,d). Next, cell supernatants were collected and extracellular secretion of IL-1β and TNF-α proteins were detected by ELISA. Similarly, increased IL-1β and TNF-α secretion was observed upon TUC339 knockdown with or without LPS stimulation (Figure 4e,f). Increased IL-1β secretion was also detected upon lincRNA-VLDLR knockdown using ELISA but with less amplitude (Figure S1c). We, thus, focus our functional study on TUC339 in the following study.

Enhanced pro-inflammatory cytokine production indicates TUC339 depletion and promotes activation of macrophages. We then asked the effect of TUC339 knockdown on expression of co-stimulatory molecule CD86 in macrophage cells. Consistent with the above results, increased CD86 expression was found in THP-1 cells upon TUC339 knockdown both with or without LPS stimulation (Figure 4g).

Next, we evaluated the potential effect of TUC339 knockdown on phagocytosis capability of THP-1 cells using the phagocytosis assay kit. Even though the phagocytosis capacity of THP-1 cells was little affected by siRNAs without LPS induction. Knockdown of TUC339 significantly enhanced phagocytosis in THP-1 cells upon LPS stimulation (Figure 4h). However, the opposite effect was observed with lincRNA-VLDLR knockdown (Figure S1d).

Lastly, we asked the effect of TUC339 knockdown on THP-1 cell viability. THP-1 cells were first transfected with either TUC339 siRNA or control siRNA and challenged by LPS stimulation. Then the CCK-8 assay was performed to determine cell viability. Our results showed that knockdown of TUC339 significantly reduced the viability of THP-1 cells (Figure 4i). Similar results were observed with lincRNA-VLDLR knockdown (Figure S1e).

To sum up, our loss-of-function experiments strongly suggest a critical role of TUC339 in fine-tuning macrophage activation in THP-1 cells. Depletion of TUC339 results in increased pro-inflammatory cytokine production, increased co-stimulatory molecule expression, enhanced phagocytosis, and reduced viability.

### 2.5. Over-Expression of TUC339 in THP-1 Cells Leads to Reduced Pro-Inflammatory Cytokine Production, Decreased co-Stimulatory Molecule Expression, and Compromised Phagocytosis

**Figure 5:**
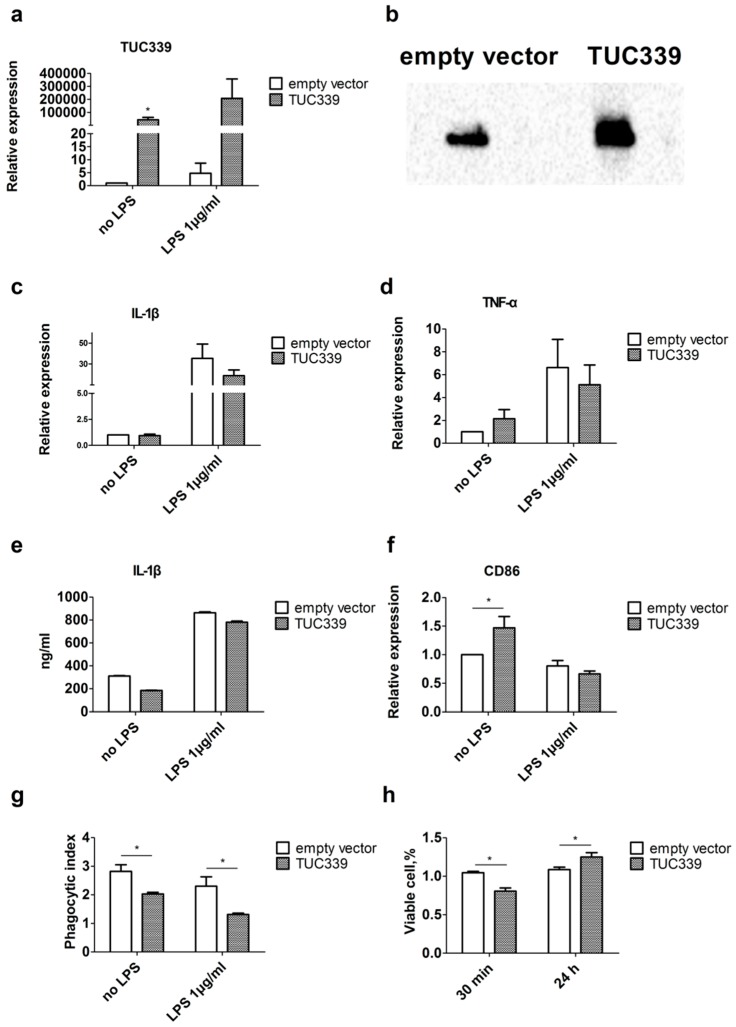
Over-expression of TUC339 in THP-1 cells leads to reduced pro-inflammatory cytokine production, decreased co-stimulatory molecule expression, and compromised phagocytosis. THP-1 cells were transfected with TUC339 expression vector or empty control vector. Both (**a**) qRT-PCR and (**b**) Northern blot analysis showed effective over-expression of TUC339. Upon TUC339 over-expression, IL-1β (**c**) and TNF-α (**d**) mRNAs were reduced in THP-1 cells with LPS stimulation as shown by qRT-PCR. (**e**) IL-1β secretion was reduced as shown by ELISA. (**f**) CD86 mRNA was reduced in THP-1 cells with LPS stimulation, which is shown by qRT-PCR. (**g**) Phagocytosis was compromised in THP-1 cells upon TUC339 over-expression. (**h**) Cell viability was increased in THP-1 cells upon TUC339 over-expression at 24 h, which is shown by a CCK-8 assay. Data are representative of three separate experiments and show the means ± SEM. * *p* < 0.05.

In order to study the effect of TUC339 over-expression, we first got a TUC339 full-length clone in pTracer-CMV2 expression vector. The TUC339 expression vector or the empty control vector were transfected into THP-1 cells, respectively. Then the TUC339 expression level in THP-1 cells were determined by both qRT-PCR and Northern blotting. A dramatic increase in TUC339 expression was observed in THP-1 cells upon transfection with the TUC339 expression vector when compared to the empty control vector (Figure 5a,b).

We then asked what would be the effect of TUC339 over-expression on environmental macrophages. We, thus, did similar functional experiments as loss-of-function studies. THP-1 cells were first transfected with either TUC339 expression vector or empty control vector and subjected to LPS stimulation. Production of pro-inflammatory cytokines IL-1β and TNF-α were measured by qRT-PCR and ELISA. Expression of co-stimulatory molecule CD86 was detected by qRT-PCR. Phagocytosis capability of THP-1 cells was determined using a phagocytosis assay kit. In addition, viability of THP-1 cells was evaluated by the CCK-8 assay. We found that, compared to an empty control vector, TUC339 over-expression in THP-1 cells results in a slight decrease in IL-1β and TNF-α production upon LPS stimulation at mRNA and the protein level (Figure 5c–e). TUC339 over-expression in THP-1 cells also results in decreased CD86 expression upon LPS stimulation (Figure 5f), declined phagocytosis (Figure 5g), and slightly increased viability at 24 h (Figure 5h). Thus, all the data merge to suggest that the effect of TUC339 over-expression generally mirrored that of TUC339 knockdown. TUC339 in the THP-1 cell plays an important role in regulating macrophage activation.

### 2.6. TUC339 is Required for M(IL-4) Macrophages Polarization

**Figure 6:**
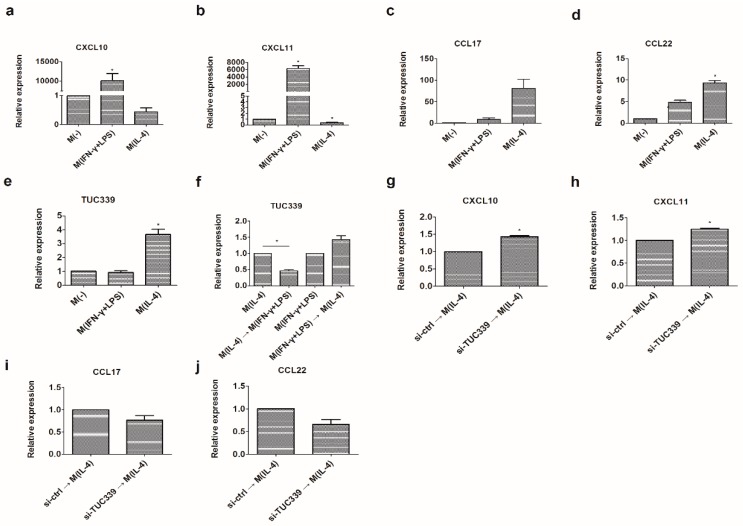
TUC339 promotes the THP-1 macrophages transition to the M(IL-4) phenotype. THP-1 macrophage cells were cultured in the presence of IFN-γ (20 ng/mL) plus LPS (100 ng/mL) or in IL-4 (20 ng/mL). Polarization-specific biomarkers were analyzed by qRT-PCR assays using RNA collected from macrophages at 18 h post-treatment. CXCL10 (**a**) and CXCL11 (**b**) were elevated in M(IFN-γ + LPS), CCL17 (**c**) and CCL22 (**d**) were elevated in M(IL-4). (**e**) Expression of TUC339 was elevated in M(IL-4) macrophages. (**f**) Differential expression of TUC339 during macrophage re-polarization. (**g**–**j**) Macrophages were transfected with siRNA-TUC339 or unrelated siRNAs control and then stimulated with IL-4 for M(IL-4) polarization. Knockdown of TUC339 increased the expression of M1 maker CXCL10 (**g**) and CXCL11 (**h**) and decreased expression of the M2 maker CCL17 (**i**) and CCL22 (**j**). Data are representative of three separate experiments and show the means ± SEM. * *p* < 0.05.

Macrophages can be polarized to M1 or M2 subgroup upon activation by environmental stimulator. We wondered whether TUC339 is involved in balancing M1/M2 polarization. In order to test this, we first generated M(IFN-γ + LPS) and M(IL-4) macrophages in vitro by incubating THP-1 cells with corresponding stimulators. M1/M2 phenotypic and functional discriminating markers such as CXCL10, CXCL11, CCL17, and CCL22 were examined using qRT-PCR. As expected, M1-associated markers CXCL10 and CXCL11 were significantly up-regulated in M(IFN-γ + LPS) (Figure 6a,b) while M2-associated markers CCL17 and CCL22 were significantly up-regulated in M(IL-4) (Figure 6c,d). These results confirmed that distinct macrophage subgroups were developed by the polarization conditions used in this study.

Next, we asked whether TUC339 expression alteration is associated with M(IFN-γ + LPS) and M(IL-4) polarization. qRT-PCR result shows that, comparing to non-polarizing control, TUC339 was significantly down-regulated in M(IFN-γ + LPS) while dramatically up-regulated in M(IL-4) macrophage (Figure 6e). Moreover, when M(IL-4) or M(IFN-γ + LPS) was re-polarized by IFN-γ + LPS or IL-4 correspondingly for 18 h, the level of TUC339 was strikingly decreased during M(IL-4)-to-M(IFN-γ + LPS) re-polarization while increased during M(IFN-γ + LPS)-to-M(IL-4) re-polarization (Figure 6f). These data suggest TUC339 expression is positively related to the M(IL-4) subgroup while negatively related to the M(IFN-γ + LPS) subgroup. This expression profile is in line with the functional study of TUC339 above.

We then asked whether TUC339 expression is required for M(IFN-γ + LPS) and M(IL-4) polarization. THP-1 cells were transfected with TUC339 siRNA or control siRNA for 48 h and then were treated with IL-4 for 18 h. Again M1/M2 phenotypic and functional discriminating markers CXCL10, CXCL11, CCL17, and CCL22 were examined using qRT-PCR. Compared to a control, depletion of TUC339 yielded enhanced CXCL10 and CXCL11 expression (Figure 6g,h) and yet reduced CCL17 and CCL22 expression, which indicated that the M(IL-4) polarization by IL-4 was partially inhibited (Figure 6i,j). Altogether, our data strongly suggest that TUC339 is required for M(IL-4) polarization.

**Figure 7:**
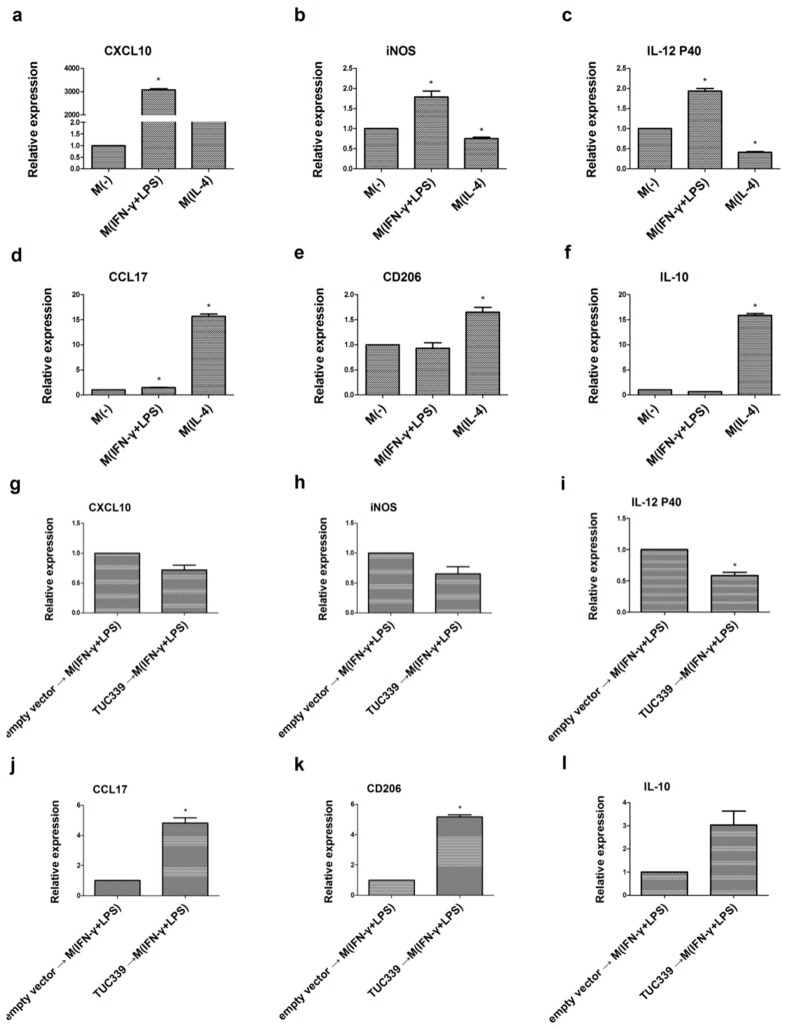
TUC339 promotes U937 macrophages transition to M(IL-4) phenotype. U937 macrophage cells were cultured in the presence of IFN-γ (20 ng/mL) plus LPS (100 ng/mL) or in IL-4 (20 ng/mL). Polarization-specific biomarkers were analyzed by qRT-PCR assays using RNA collected from macrophages 18 h post-treatment. As expected, CXCL10 (**a**) iNOS (**b**) and IL-12 p40 (**c**) were elevated in M(IFN-γ + LPS) while CCL17 (**d**) CD206 (**e**) and IL-10 (**f**) were elevated in M(IL-4). (**g**–**l**) Macrophages were transfected with the TUC339 expression vector or the empty control vector and then stimulated with IFN-γ + LPS for M1 polarization. Over-expression of TUC339 decreased expression of M1 maker CXCL10 (**g**) iNOS (**h**) and IL-12 p40 (**i**) and increased expression of M2 maker CCL17 (**j**) CD206 (**k**) and IL-10 (**l**). Data are representative of three separate experiments and show the means ± SEM. * *p* < 0.05.

In order to rule out the possibility that the effect of TUC339 on macrophage polarization is THP-1 cell line specific, we conducted the experiment with a second macrophage cell line U937. The same M(IFN-γ + LPS) and M(IL-4) polarization conditions worked for U937 cells, which was verified by corresponding M1/M2 marker expression (Figure 7a–f). We then examined the effect of TUC339 over-expression on U937 M1/M2 polarization. We transfected U937 cells with either a TUC339 expression vector or an empty control vector for 48 h and then treated the cells with IFN-γ + LPS for 18 h. M1/M2 discriminating markers were again examined using qRT-PCR. As seen in Figure 7g–l, comparing to a control, over-expression of TUC339 yielded significantly reduced IL-12 p40 expression (M1 marker), while significantly enhanced CCL17 and CD206 expression (M2 markers). This result suggests that TUC339 promotes M(IL-4) polarization in U937 cells, which is again consistent with our previous findings. This points toward TUC339 driving M(IL-4) polarization in the macrophage.

### 2.7. Transcriptome-Wide Analysis Reveals Pathways Downstream of TUC339

**Figure 8:**
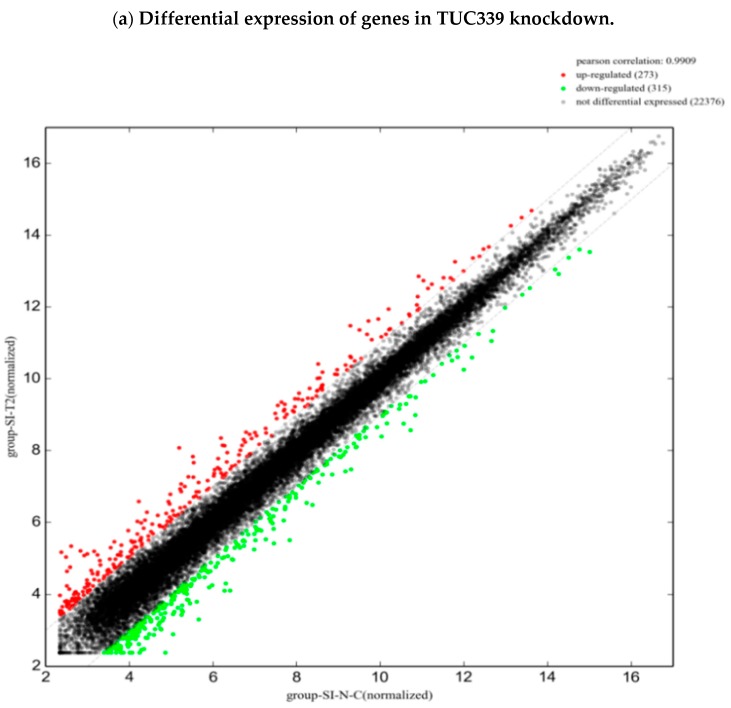

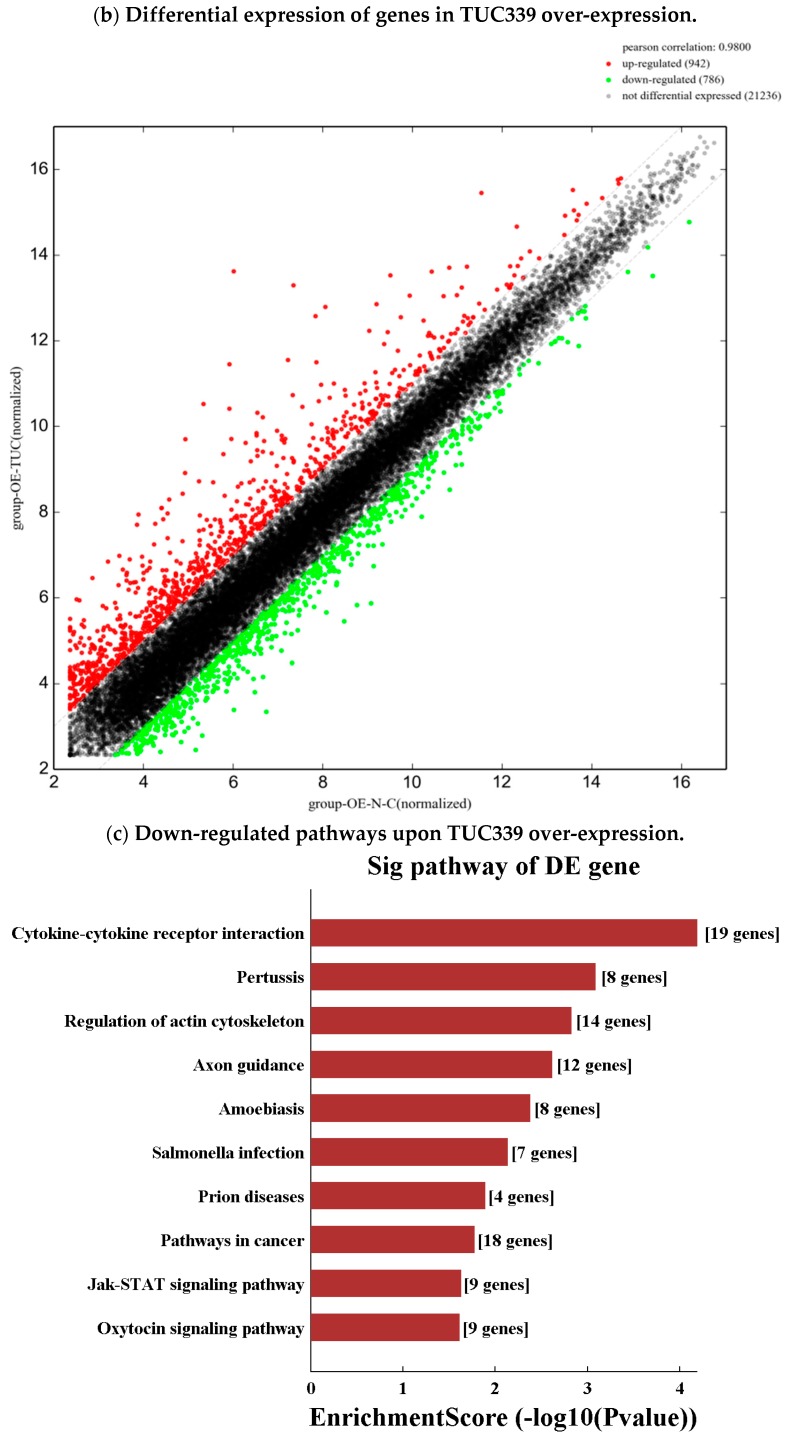

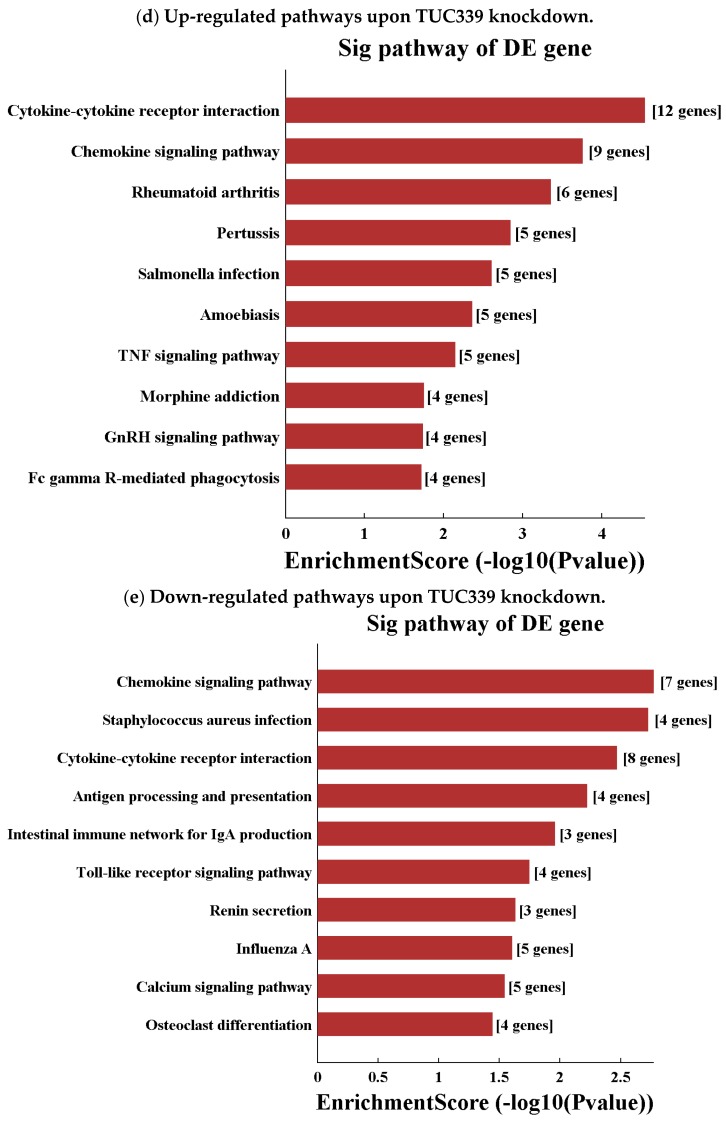

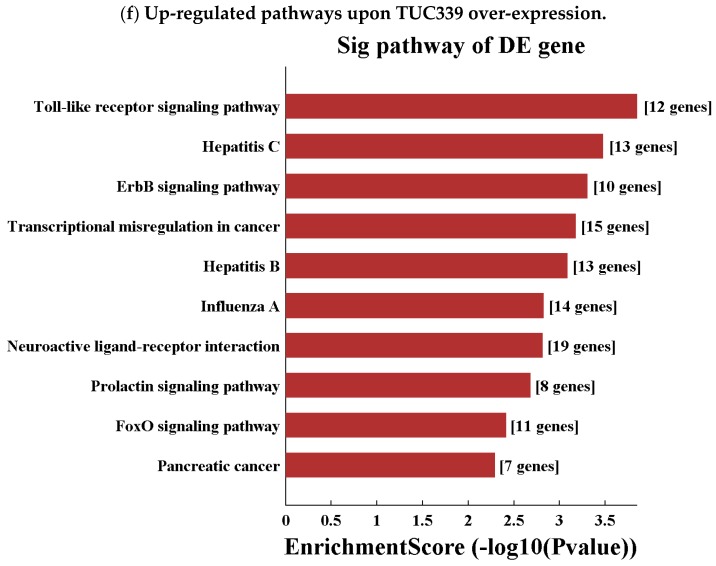
Transcriptome-wide analysis of TUC339 knockdown and over-expression. Differential expressions of genes are plotted (axis, intensity). (**a**) 273 up-regulated genes and 315 down-regulated genes with greater than a two-fold change upon TUC339 knockdown. (**b**) 942 up-regulated genes and 786 down-regulated genes with greater than a two-fold change upon TUC339 over-expression. (**c**) Down-regulated pathways upon TUC339 over-expression. (**d**) Up-regulated pathways upon TUC339 knockdown. (**e**) Down-regulated pathway upon TUC339 knockdown. (**f**) Up-regulated pathways upon TUC339 over-expression. The bar plot shows the top 10 Enrichment Score (−log10 (*p* value)) values of the significant enrichment pathways.

To study the transcriptome-wide impact of TUC339 in macrophages, we performed microarray analysis on LPS challenged THP-1 cells upon TUC339 over-expression or knockdown. Comparing the TUC339 knockdown group to the control siRNA group, we identified 273 up-regulated genes and 315 down-regulated genes with greater than a two-fold change (Figure 8a). Comparing the TUC339 over-expression group to an empty vector control group, 942 up-regulated and 786 down-regulated genes with greater than two-fold change were identified (Figure 8b). GO analysis of these differential expressed genes revealed a significant enrichment of genes involved in CXCR chemokine receptor binding, cytokine signaling pathway, cell proliferation, an immune system defense response, and regulation of a response to stimulus (Figure S2). The top 10 most significantly altered pathways were also analyzed (Figure 8c–f). Many up-regulated pathways from the over-expression experiment overlap with those down-regulated from the knockdown experiment and vice versa. Pathways of cytokine-cytokine receptor interaction, viral and bacterial infections, Toll-like receptor signaling, FcγR-mediated phagocytosis, and regulation of the actin cytoskeleton were highlighted. Microarray data are generally in line with our findings that TUC339 modulates macrophage activation and M1/M2 polarization.

## 3. Discussion

In this study, we identified lncRNA TUC339 as a novel signaling mediator that is enriched in HCC-derived exosomes and plays important roles in macrophage activation and M1/M2 polarization regulation. We showed that over-expression of TUC339 in THP-1 cells resulted in reduced pro-inflammatory cytokine production, decreased co-stimulatory molecule expression, and compromised phagocytosis and vice versa. Moreover, TUC339 expression was positively associated with M(IL-4) macrophages polarization. Blockade of TUC339 expression in macrophages inhibited M(IL-4) polarization while excess TUC339 expression in macrophages promoted M(IL-4) polarization. Our results unveil a novel function of HCC-derived exosomal lncRNA in the tumor microenvironment and a novel mechanism of tumor-immune cells interaction.

Previously, lncRNA TUC339 was identified as a lncRNA that is enriched in HCC-derived exosomes and transfers between HCC cells to promote tumor growth and spread [18]. In this scenario, we provide evidence to suggest TUC339 as a novel signaling mediator, which can be transferred from HCC tumor cells to environmental macrophages and subsequently dampen the immune response to tumor cells. As the tumor progresses, tumor cells develop a variety of strategies to evade immune surveillance and interrupt the immune attack [21]. For example, tumor-derived exosomes are enriched for FasL and TRAIL, which can promote T-cell apoptosis [22,23]. Along with these findings, our result provides important new insights into tumor-immune interaction and uncovers potential therapeutic targets for treatment of HCC.

Phagocytosis is a major feature of macrophages that is meant to engulf and clear external microbes, dead or damaged cells to provide an effective immune defense, and maintain homeostasis of the organism [24]. Phagocytosis can be effectively triggered by a toll-like receptor (TLR), a Fcγ receptor (FcγR), and complement receptor (CR) mediated ligand binding. FcγR facilitates the phagocytosis of particles that are opsonized by antibodies. In our microarray study, we showed that the FcγR-mediated phagocytosis pathway was up-regulated upon knockdown of TUC339, which may partially explain the enhanced phagocytosis observed in TUC339 knockdown. As known, the actin cytoskeleton, which maintains cellular morphology, is the primary force driving phagocytosis [25]. We observed a down-regulation of the actin cytoskeleton pathway upon the over-expression of TUC339, which is in line with down-regulated phagocytosis under that condition. Moreover, GO analysis of the microarray data indicates the cell motility and cell migration of THP-1 cells, which were suppressed upon over-expression of TUC339. Further exploration on direct downstream targets of TUC339 and more detailed mechanistic analyses on TUC339 effect on phagocytosis are required in future studies.

Inflammation is a defensive response of our immune system against microbial invasion or tissue damage [26]. LPS-induced inflammation promotes the classical activation pathway of macrophages toward M1 phenotype (pro-inflammatory) through TLR (toll-like receptor) stimulation, which results in pro-inflammatory cytokines production such as TNF-α, IL-1β, IL-6, IL-12, and IL-23. In this case, upon TUC339 knockdown, we observed increased pro-inflammatory cytokine production, enhanced phagocytosis, and elevated co-stimulatory molecule expression by THP-1 cells, which suggests classical activation of macrophage. Over-expression of TUC339, in contrast, led to reduced pro-inflammatory cytokine production, compromised phagocytosis, and decreased co-stimulatory molecule expression by THP-1 cells. These observations are consistent with each other and lead us to examine the macrophage polarization status upon alteration of TUC339 expression.

Macrophages can be polarized to pro-inflammatory M(IFN-γ + LPS) or anti-inflammatory M(IL-4) status. These two distinct subgroups can be dynamically converted into each other under a specific microenvironment [27]. Previous studies revealed a significantly altered lncRNA and mRNA expression profile in macrophages exposing to different activating conditions, which indicated that lncRNAs may play important roles in regulating macrophage polarization. LncRNA TCONS_00019715 was found to play an important role in promoting macrophage polarization to the M(IFN-γ + LPS) phenotype [28]. LncRNA cox-2 can inhibit tumor growth by inhibiting the polarization of M2 macrophages [29]. Exosomes have also emerged as important signaling mediators of macrophage polarization. LPS-preconditioned mesenchymal stromal cells-derived exosomes induced up-regulation of anti-inflammatory cytokines and M2 macrophage activation [30]. Tumor-derived exosomal miRNA can be transferred to tumor-associated macrophages and stimulate polarization towards the M2 phenotype to promote tumor progress [31]. In this study, to examine whether TUC339 contributes to the plasticity of macrophage polarization, we measured the expression of TUC339 in M(IFN-γ + LPS) and M(IL-4)-polarized activation of macrophages and found that M(IL-4) macrophages exhibited a considerably higher level of TUC339 than control and M(IFN-γ + LPS) macrophages. Next, we examined differential expression of TUC339 during macrophage polarization by incubating M(IFN-γ + LPS) macrophages with IL-4 and M(IL-4) macrophages with IFN-γ + LPS. Our experiments demonstrated that M(IFN-γ + LPS)-to-M(IL-4) conversion resulted in increased TUC339 expression while M(IL-4)-to-M(IFN-γ + LPS) led to decreased TUC339 expression. Lastly, it was very interesting to see that knockdown of TUC339 stimulated M(IL-4)-to-M(IFN-γ + LPS) transition (Figure 6g–j) while over-expression of TUC339 stimulated M(IFN-γ + LPS)-to-M(IL-4) transition (Figure 7g–l). Notably, both THP-1 and U937 human macrophage cell lines were tested and similar conclusions were drawn, thus arguing against the possibility that the regulatory effect of TUC339 was cell line-specific. Taken together, our data suggest that exosomal TUC339 plays an important role in promoting macrophage polarization to the M(IL-4) phenotype. From our microarray data, we found that TUC339 is involved in cytokine receptor signaling pathways and CXCR chemokine receptor binding pathways, which may provide some clues on the underlining mechanisms of this regulation even though the exact details are worth further investigation.

In summary, the data presented in this study demonstrate a novel role for HCC-derived exosomal lncRNA TUC339 in macrophage activation and M1/M2 polarization. Our study not only advanced our understanding of lncRNA on regulating macrophage function but also shed light on the complicated interactions between tumor cells and innate immunity.

## 4. Materials and Methods

### 4.1. Cell Culture

THP-1, U937, and HL-7702 cells (ZSGB-BIO, Beijing, China) were cultured in RPMI-1640 medium (HyClone Laboratories, Logan, UT, USA) containing 10% heat-inactivated fetal bovine serum (FBS) (Thermo Fisher Scientific, Waltham, MA, USA), 100 U/mL penicillin, and 100 μg/mL streptomycin (HyClone Laboratories, Logan, UT, USA). Cells were cultured at 37 °C in a humidified incubator in which the concentration of CO_2_ was 5% and were used in the exponential growth phase.

### 4.2. Isolation of Exosomes Derived from PLC/PRF/5 Cells

PLC/PRF/5 cells (1 × 10^6^/well) were plated in vesicle-depleted medium for 2 days prior to the collection of exosomes. The medium was first centrifuged at 300× *g* for 10 min and then at 2000× *g* for 20 min at 4 °C to remove cells and then at 10,000× *g* for 20 minutes at 4 °C. The supernatant was then centrifuged at 100,000× *g* for 70 min at 4 °C to pellet exosomes, which were then washed by resuspending in phosphate-buffered saline (PBS) and ultra-centrifuged at 100,000× *g* for 70 min 4 °C. The final pellet was re-suspended with 50 to 100 uL PBS and stored at 4 °C or −80 °C.

### 4.3. Transmission Electron Microscopy

Pellets of exosomes were added to carbon film copper network and adsorbed 5 min to dry up. Then copper network was stained with phosphotungstic acid for 3 min and blotted with paper until air dried. The images were obtained using FEI Technai G2F20S-Twin filed-emission transmission electron microscopy (FEI-TEM).

### 4.4. Nanoparticle Tracking Analysis

The size distribution analysis of exosomes was determined by the Shanghai XP Biomed Ltd. using an Electrophoresis & Brownian Motion Video Analysis Laser Scattering Microscopy (Zataview, Particle Metrix, Germany). The capture settings and analysis settings were performed manually, according to the manufacturer’s instructions.

### 4.5. Western Blot

Cells were lysed with RIPA buffer (Sigma, St. Louis, MO, USA). Isolated exosomes were lysed in 5× protein loading buffer (Beyotime, Shanghai, China). Equivalent amounts of cell lysate and exosomes were subjected to 10% SDS-polyacrylamide gel and transferred to PVDF membrane (Millipore, Burlington, MA, USA). After being blocked with 5% skim milk for 1 h at room temperature, the membrane was probed with the following primary antibodies: CD63 (Abcam, Cambridge, MA, USA, 1:2000), β-Actin (Abcam, 1:2500), and the HRP-conjugated secondary antibody against rabbit (Abcam, Cambridge, MA, USA, 1:5000). The blot was visualized by enhanced chemiluminescence using ChemiDoc XRS system (Bio-Rad Laboratories, Hercules, CA, USA).

### 4.6. Cellular Internalization of PLC/PRF/5 Cells-Derived Exosomes

PLC/PRF/5 cells-derived exosomes were labeled with PKH67 Green Fluorescent Cell Linker Mini Kit (Sigma-Aldrich, St. Louis, MO, USA), according to the manufacturer’s protocol with minor modifications. Exosomes diluted in PBS were added to 100 μL Diluent C. In parallel, 0.5 μL PKH67 dye was added to 100 μL Diluent C and incubated with exosomes solution for 4 min. To bind excess dye, 200 μL 1% BSA/PBS was added. The labeled exosomes were filtered using 0.22 μm filter to remove excess dye and added onto THP-1 cells cultured in a Thermo Scientific eight-chamber slide. After incubation for 24 h, cells were stained with Hoechst 33258 (Sigma-Aldrich, St. Louis, MO, USA) and examined by confocal fluorescence microscopy.

### 4.7. Macrophage-Differentiation and Polarization Conditions

THP-1 or U937 cells were treated with 100 ng/mL phorbol-12-myristate-13-acetate (PMA) (Sigma-Aldrich, St. Louis, MO, USA) overnight. To induce the polarization of macrophages, PMA-differentiated THP-1 cells were treated with 20 ng/mL of IFN-γ and 100 ng/mL of LPS (Sigma-Aldrich, St. Louis, MO, USA) to achieve M(IFN-γ + LPS) polarization or with 20 ng/mL of IL-4 (PeproTech, Rocky Hill, NJ, USA) to achieve M(IL-4) polarization. The non-polarizing THP-1 macrophages were cultured and left untreated. After 18 h of polarization, the adherent cells were harvested for further analysis.

### 4.8. Phagocytosis Assay

THP-1 cells (1 × 10^6^/well) were plated in six-well culture plates and allowed to adhere overnight. The Latex beads-rabbit IgG-FITC complex (Cayman, Ann Arbor, MI, USA) was added directly to the culture medium at a 1:200 dilution and incubated at 37 °C for two hours. Cells were gently washed with assay buffer twice, which was followed by visualization.

### 4.9. CCK-8 Assay

For cell viability studies, THP-1 cells were seeded (1 × 10^4^/well) in collagen-coated 96-well plates in the appropriate media and incubated for 24 h. Then medium was replaced with medium containing 100 ng/mL PMA and incubated for 24 h. After being transfected with siRNA or plasmid for 48 h, THP-1 cells were treated with 1 μg/mL LPS for 24 h. The viability was assessed using CCK-8 solution (Dojindo Molecular Technologies, Rockville, MD, USA) and a microplate reader (Bio-Rad Laboratories, Hercules, CA, USA).

### 4.10. Real-Time PCR

Total RNAs were extracted from cells using Trizol (Thermo Fisher Scientific, Waltham, MA, USA) and 1 μg of RNA was reverse-transcribed to cDNA using iScript cDNA Synthesis Kit (Bio-Rad Laboratories, Hercules, CA, USA). The expression of genes was quantified using SYBR green reagents (Life Technologies, Carlsbad, CA, USA) on a CFX96 touch real time PCR detection system (Bio-Rad Laboratories, Hercules, CA, USA) using primers indicated in Supplementary Table S1.

### 4.11. Transfection of siRNA

Three independent siRNAs against TUC339 and lincRNA-VLDLR along with unrelated control siRNA (si-ctrl) were purchased from Ribobio (Guangzhou, China). THP-1 cells were transfected with 50 nM siRNA to lncRNA or si-ctrl using INTERFERin (Polyplus-transfection SA, Illkirch-Graffenstaden, France) for 48 h before further experiments. si-TUC339-1 was used in functional studies.

### 4.12. ELISA

The levels of TNF-α and IL-1β in culture supernatants were determined by using ELISA kits (Wuhan Boster Bio-Engineering, Wuhan, China), according to the manufacturer’s instructions. The levels of TNF-α and IL-1β were quantified using a standard curve established by a serial dilution of standard concentration.

### 4.13. Northern Blot

A specific 5′-biotinylated probe to detect TUC339 was designed and synthesized by Tsingke (Chengdu, China) with the sequence shown below: 5′-GGATCGGTGTGAAATAACGGGCCCATATAAATCCC-3′. Additionally, 10 μg total RNAs were separated on 6% Urea PAGE gel and were transferred to a nylon membrane. Northern blotting was performed with the Chemiluminescent Nucleic Acid Detection Module (Thermo scientific, Waltham, MA, USA). The probed membrane was washed twice with 2 × SSC (300 mM NaCl, 30 mM sodium citrate, pH 7.0) with 0.1% SDS for 10 min at room temperature and with 0.1 × SSC with 0.1% SDS for 5 min. HRP-conjugated anti-digoxigenin was used to detect the hybridized probe by chemiluminescent imaging with a lumino-image analyzer.

### 4.14. Gene Over-Expression

Full length of TUC339 was cloned into the pTracer-CMV2 vector (Jingmai BioTech, Chengdu, China). Differentiated THP-1 cells were transfected with 2 μg of TUC339 expressing vector or empty control vector using Jetprime (Polyplus-transfection SA, France) and, after 48 h, the cells were used for further experiments.

### 4.15. Genome-Wide Gene Expression Analysis

THP-1 differentiated cells were transfected with either si-TUC339, si-ctrl, TUC339 expression vector, or empty control vector for 48 h and were then treated with 1 μg/mL LPS for 16 h. The harvested cells were used for RNA isolation and microarray analysis by Kangchen Bio-tech Inc. (Shanghai, China) using Agilent human 4 × 44 k Gene Expression Microarray V2 (Agilent Technologies, Santa Clara, CA, USA).

### 4.16. Statistical Analysis

The data were expressed as the mean and standard error from at least three replicates. Comparisons between groups were performed using the two-tailed Student’s t test and results were considered to be statistically significant when *p* < 0.05.

## Supplementary Materials

Supplementary materials can be found at http://www.mdpi.com/1422-0067/19/10/2958/s1.
