# Supplementary material for: Regulation of Macrophage Activation and Polarization by HCC-Derived Exosomal lncRNA TUC339
Source: Int J Mol Sci. 2018 Sep 28;19(10):2958. doi: 10.3390/ijms19102958 (PMC6213212; doi:10.3390/ijms19102958)
Supplement: Supplementary file 1 [file ijms-19-02958-s001.pdf]

Supplementary Table 1. RT-qPCR primers and siRNA sequences

Name	sequences
qPCR primers	
U6	Sense 5'-CTCGCTTCGGCAGCACA-3'
	Anti-sense 5'-AACGCTTCACGAATTTGCGT-3'
linc-VLDLR	Sense 5'-AGCAGTCACATTCATCGCAC-3'
	Anti-sense 5'-GAGGAATAGGTGCGAACTGC-3'
TUC339	Sense 5'-GATGAGGCCCCGAGTTTAAT-3'
	Anti-sense 5'-AGATGGAGGATCGGTGTGAA-3'
CD86	Sense 5'-ATTCCAAGTATATGGGCCGC-3'
	Anti-sense 5'-TGTGGGCTTTTTGTGATGGA-3'
TNF-α	Sense 5'-GGAGAAGGGTGACCGACTCA -3'
	Anti-sense 5'-CTGCCCAGACTCGGCAA -3'
IL-1β	Sense 5'-ACAGATGAAGTGCTCCTTCCA-3'
	Anti-sense 5'-GTCGGAGATTCGTAGCTGGAT-3'
CXCL10	Sense 5'-GGCCATCAAGAATTTACTGAAAGCA-3'
	Anti-sense 5'-TCTGTGTGGTCCATCCTTGGAA-3'
CXCL11	Sense 5'-CCTTGGCTGTGATATTGTGTGCTA-3'
	Anti-sense 5'-CCTATGCAAAGACAGCGTCCTC-3'
CCL17	Sense 5'-TGAGGACTGCTCCAGGGATG-3'
	Anti-sense 5'-AACGGTGGAGGTCCCAGGTA-3'
CCL22	Sense 5'-GAGACATACAGGACAGAGCATGGC-3'
	Anti-sense 5'-ATGGAGATCAGGGAATGCAGAGAG -3'
iNOS	Sense 5'-CAGCGGGATGACTTTCCAA-3'
	Anti-sense 5'-AGGCAAGATTTGGACCTGCA-3'
IL-12p40	Sense 5'-TGAAGAAAGATGTTTATGTCGTAGAAT-3'
	Anti-sense 5'-GGTCCAAGGTCCAGGTGATA-3'
CD206	Sense 5'-CGGTGACCTCACAAGTATCCACAC-3'
	Anti-sense 5'-TTCATCACCACACAATCCTCCTGT-3'
IL-10	Sense 5'-GGTTGCCAAGCCTTGTCTGA-3'
	Anti-sense 5'-AGGGAGTTCACATGCGCCT-3'

siRNA	
linc-VLDLR	siRNA1 5’-CCCTGGATCTACAGGTCAT-3’
	siRNA2 5’-CCCTTAGCTGAATCATGTT-3’
	siRNA3 5’-GCCAATAAAGTTGTCCCTA-3’
TUC339	siRNA1 5’-CCTCCTATGTAAATTGAGA-3’
	siRNA2 5’-GCGACAATTTAGACACTCA-3’
	siRNA3 5’-GGAATTTCAATGCGGCCAA-3’

Supplementary figure legend

Fig S1. Enrichment of linc-VLDLR in PLC/PRF/5-derived exosomes and functional study of linc-VLDLR in THP-1 cells. (a) Linc-VLDLR was enriched in PLC/PRF/5-derived exosomes. (b) qRT-PCR showed transfection of siRNAs efficiently knocked down linc-VLDLR. (c-e) Loss-of-function studies showed depletion of linc-VLDLR led to increased IL-1β expression (c), reduced phagocytosis (d) and reduced cell viability (e). Bars represent the mean ± SEM. *, P < 0.05.

Fig S2. GO anaylsis of up and down regulated gene sets upon TUC339 knockdown and over-expression (a-d).

Supplementary figure

Fig. S1

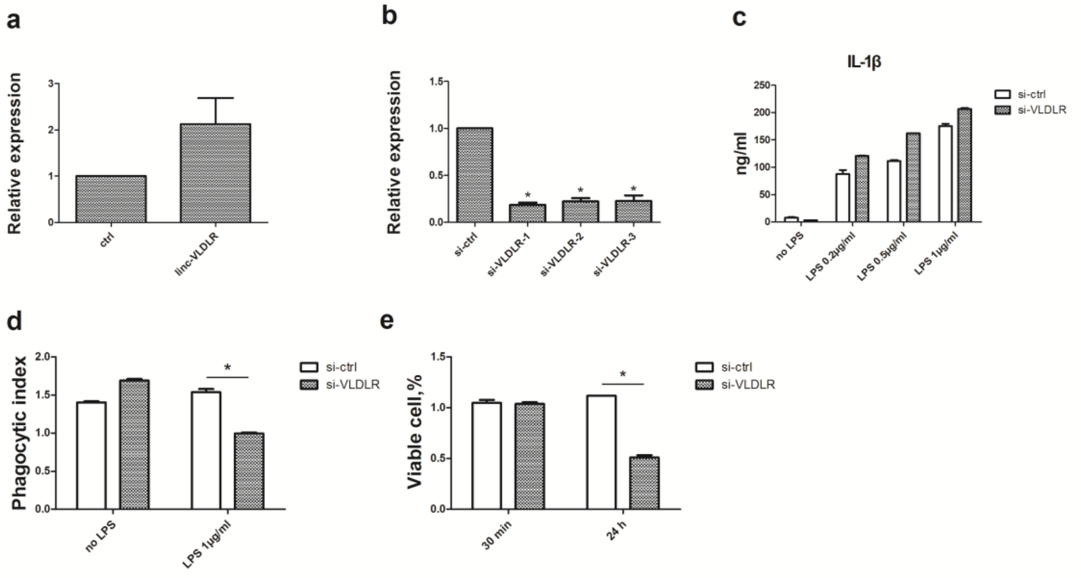

Fig. S2

a Down-regulated gene sets upon TUC339 over-expression

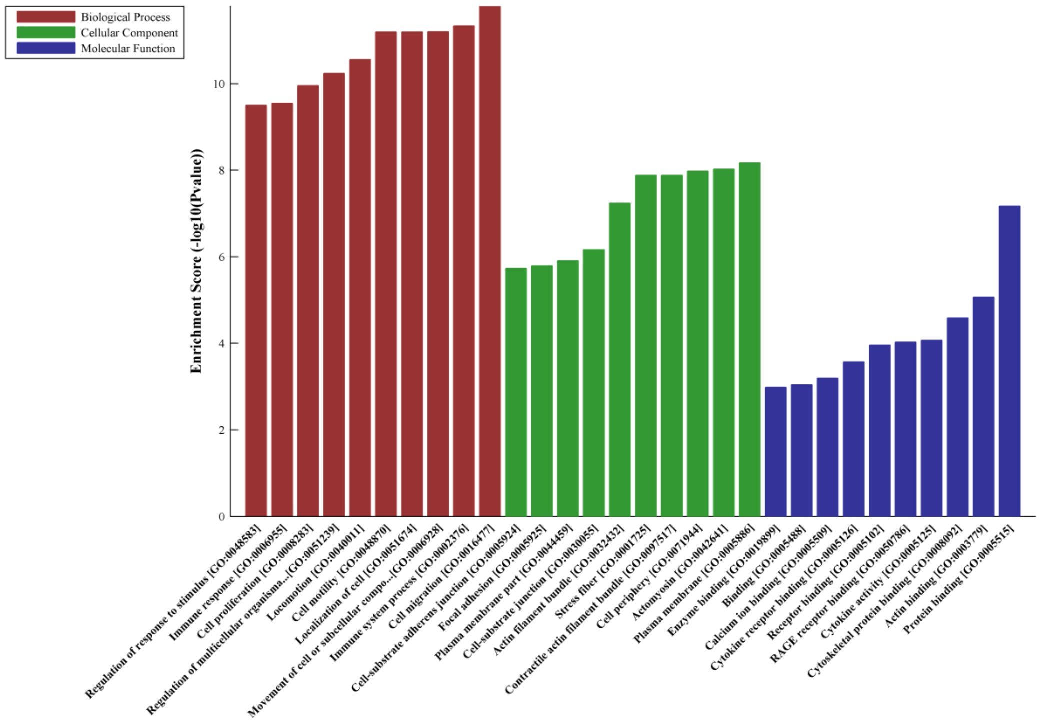

b Down-regulated gene sets upon TUC339 knockdown

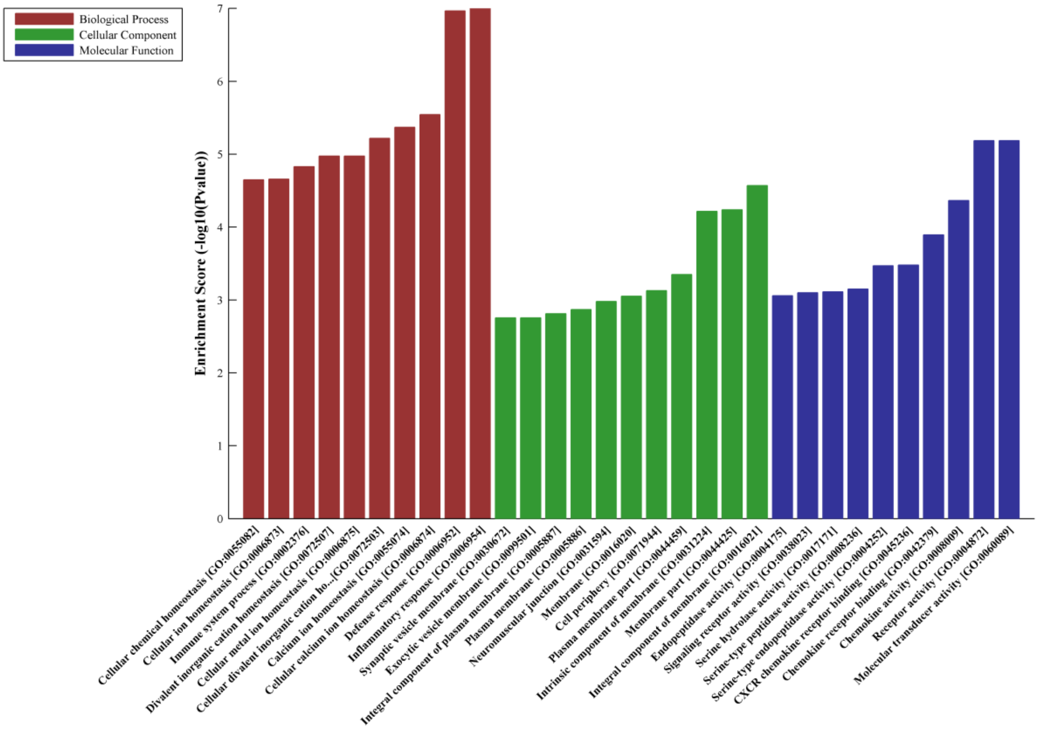

c Up-regulated gene sets upon TUC339 over-expression

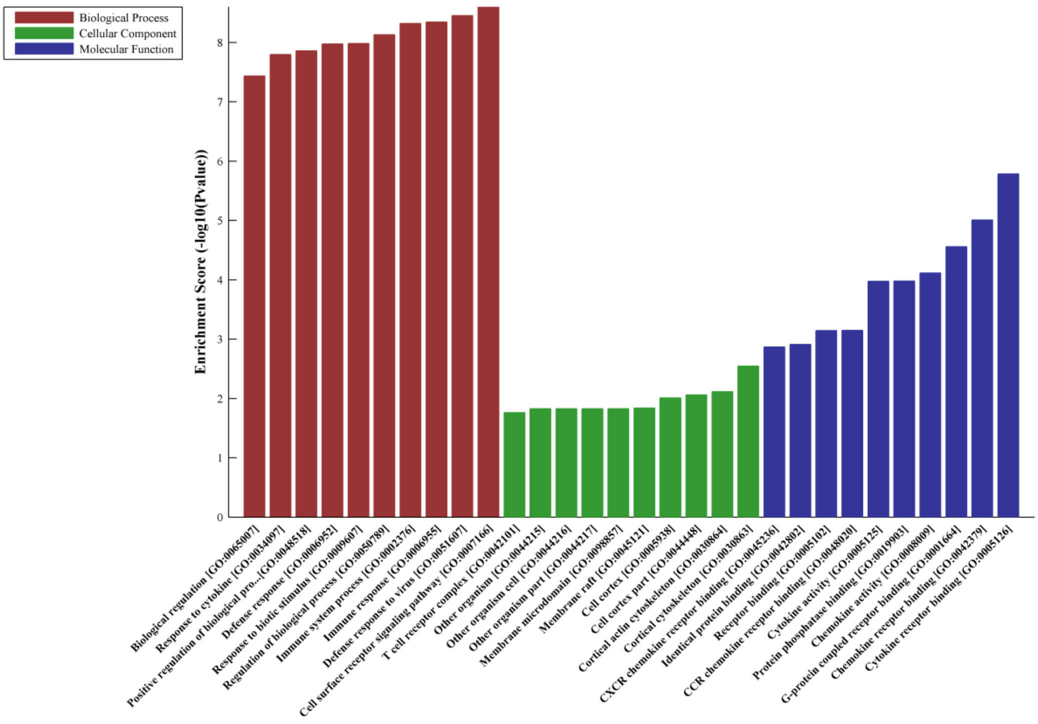

d Up-regulated gene sets upon TUC339 knockdown

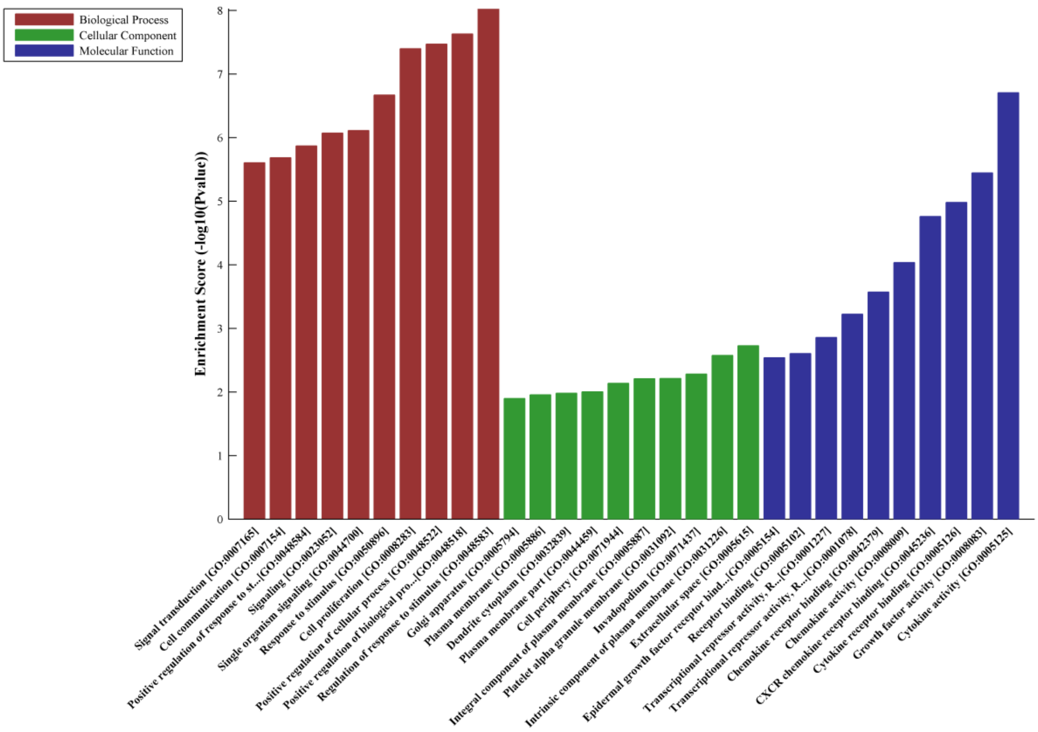
Biological Process
Cellular Component
Molecular Function
Enrichment Score (-log10(Pvalue))
0
1
2
3
4
5
6
7
8
Signal transduction [GO:0007165]
Cell communication [GO:0007154]
Positive regulation of response to st...[GO:0048584]
Signaling [GO:0023052]
Single organism signaling [GO:0044700]
Response to stimulus [GO:0050896]
Cell proliferation [GO:0008283]
Positive regulation of cellular process [GO:0048522]
Positive regulation of biological pro...[GO:0048518]
Regulation of response to stimulus [GO:0048583]
Golgi apparatus [GO:0005794]
Plasma membrane [GO:0005886]
Dendrite cytoplasm [GO:0032839]
Plasma membrane part [GO:0044459]
Cell periphery [GO:0071944]
Integral component of plasma membrane [GO:0005887]
Platelet alpha granule membrane [GO:0031092]
Invadopodium [GO:0071437]
Intrinsic component of plasma membrane [GO:0031226]
Extracellular space [GO:0005615]
Epidermal growth factor receptor bind...[GO:0005154]
Receptor binding [GO:0005102]
Transcriptional repressor activity, R...[GO:0001227]
Transcriptional repressor activity, R...[GO:0001078]
Chemokine receptor binding [GO:0042379]
Chemokine activity [GO:0008009]
CXCR chemokine receptor binding [GO:0045236]
Cytokine receptor binding [GO:0005126]
Growth factor activity [GO:0008083]
Cytokine activity [GO:0005125]
